# Food allergy and PPI-responsive esophageal eosinophilia

**DOI:** 10.1186/1939-4551-8-S1-A24

**Published:** 2015-04-08

**Authors:** David JT Huang, Jason Kangeun Ko, Jorge A Mazza

**Affiliations:** 1 Unveristy of Western Ontario, Canada; 2 Schulich School of Medicine & Dentistry, University of Western Ontario, Canada

## Background

A retrospective study to compare the food allergy prevalence in proton-pump inhibitor-responsive esophageal eosinophilia (PPI-REE) patients and patients with eosinophilic esophagitis (EoE) not responsive to PPI therapy.

## Methods

A chart review was performed for 30 patients diagnosed with EoE, prescribed PPI therapy and tested with atopic patch tests for a panel of food allergens. Patients were categorized as having PPI-REE if past clinical assessments noted significant symptomatic improvement with PPI therapy. Those without clinical response to PPI were categorized as non-responders. The two groups were compared on frequencies of other treatments offered (swallowed steroids (e.g. fluticasone, budesonide), esophageal dilatation), histology (eosinophil counts in esophageal biopsy at diagnosis) and frequency of positive food allergy tests. Statistical analysis used chi-square tests for frequency comparisons and student’s t-test for average eosinophil count comparison.

## Results

Of the 30 patients reviewed, 12 were found to have PPI-REE. There was no significant difference in other treatments offered to PPI-REE and non-responsive patients (10/12 and 8/18, respectively; p = 0.21), average eosinophil counts at diagnosis (65.7 ± 29.2 and 42.6 ± 15.6, respectively; p = 0.14), nor in likelihood of food allergy as detected by skin prick (9/12 and 9/18, respectively; p = 0.98) or food patch testing (9/12 and 9/18, respectively; p = 0.60).

## Conclusions

It was hypothesized that PPI-REE cases would be less atopic, with regards to foods, than non-responders due to the possible prevalence of undiagnosed GERD in the former group [1]. However, this review failed to show any statistically significant differences between the two groups. This is consistent with attempts of other groups to distinguish PPI-REE and EoE patients on other clinical parameters [2].
